# Peripheral Blood Monocyte Abundance Predicts Outcomes in Patients with Breast Cancer

**DOI:** 10.1158/2767-9764.CRC-22-0023

**Published:** 2022-05-04

**Authors:** Margaret L. Axelrod, Yu Wang, Yaomin Xu, Xiaopeng Sun, Cosmin A. Bejan, Paula I. Gonzalez-Ericsson, Sara Nunnery, Riley E. Bergman, Joshua Donaldson, Angel L. Guerrero-Zotano, Chiara Massa, Barbara Seliger, Melinda Sanders, Ingrid A. Mayer, Justin M. Balko

**Affiliations:** 1 Department of Medicine, Vanderbilt University Medical Center, Nashville, Tennessee.; 2 Department of Biostatistics, Vanderbilt University Medical Center, Nashville, Tennessee.; 3 Department of Biomedical Informatics, Vanderbilt University Medical Center, Nashville, Tennessee.; 4 Breast Cancer Research Program, Vanderbilt University Medical Center, Nashville, Tennessee.; 5 Medical Oncology Department, Instituto Valenciano de Oncología, Valencia, Spain.; 6 Institute of Medical Immunology, Martin Luther University Halle-Wittenberg, Halle (Saale), Germany.; 7 Department of Pathology, Microbiology and Immunology, Vanderbilt University Medical Center, Nashville, Tennessee.

## Abstract

Biomarkers of response are needed in breast cancer to stratify patients to appropriate therapies and avoid unnecessary toxicity. We used peripheral blood gene expression and cell-type abundance to identify biomarkers of response and recurrence in neoadjuvant chemotherapy–treated patients with breast cancer. We identified a signature of IFN and complement response that was higher in the blood of patients with pathologic complete response. This signature was preferentially expressed by monocytes in single-cell RNA sequencing. Monocytes are routinely measured clinically, enabling examination of clinically measured monocytes in multiple independent cohorts. We found that peripheral monocytes were higher in patients with good outcomes in four cohorts of patients with breast cancer. Blood gene expression and cell type abundance biomarkers may be useful for prognostication in breast cancer.

**Significance:**

Biomarkers are needed in breast cancer to identify patients at risk for recurrence. Blood is an attractive site for biomarker identification due to the relative ease of longitudinal sampling. Our study suggests that blood-based gene expression and cell-type abundance biomarkers may have clinical utility in breast cancer.

## Introduction

**FIGURE 1:**
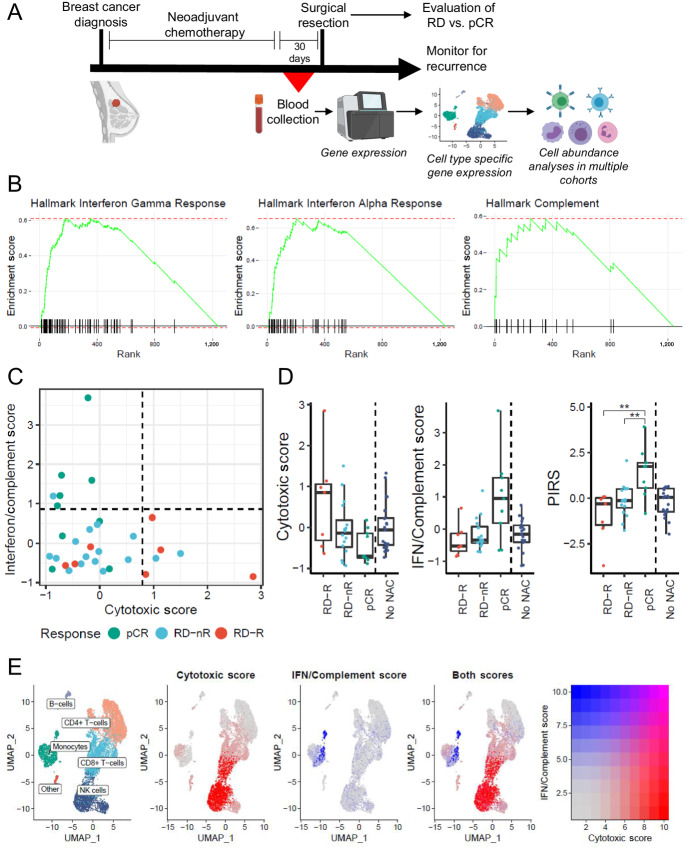
Expression of immune-related genes in the peripheral blood is associated with good outcome following NAC. **A,** Schematic describing blood collection timing and downstream analyses. **B,** GSEA plots for gene sets enriched in blood of patients with pCR relative to RD. **C,** Expression of IFN/complement and cytotoxicity scores is shown for each sample. **D,** Cytotoxic, IFN/complement, and combined PIRSs (IFN/complement score minus cytotoxic score) are shown for each sample, stratified by outcome. Box plots show the median, first and third quartiles. *P* values represent FDR-corrected Wilcoxon tests. **E,** Single-cell sequencing of PBMCs shows cell type specific expression of each score. Cytotoxic score is shown in red. IFN/complement score is shown in blue. Coexpression would be shown in pink.

Neoadjuvant chemotherapy (NAC), the standard of care for many patients with breast cancer, is known to have systemic immunologic effects and is increasingly being used in clinical trials in combination with immunotherapeutics. Currently, there are few biomarkers to predict NAC or immunotherapy response, although response to NAC is known to be associated with long-term outcome in breast cancer (1). Thus, biomarkers are needed to identify patients who will benefit from combination therapy compared with those who are likely to respond to NAC alone, and thereby avoid the added risk of toxicity and financial burden. Peripheral blood is an attractive site of biomarker development due to the relative ease of longitudinal sampling. We have previously shown that high expression of a cytotoxicity gene signature in the blood following NAC is associated with the presence of residual disease (RD) and future breast cancer recurrence, demonstrating the feasibility of using blood-based transcriptional biomarkers (2). Herein, we use peripheral blood collected following NAC (if received) and prior to surgery as a tool for biomarker development, using both gene expression and cell-type abundance analyses in multiple independent cohorts of patients with breast cancer (Fig. 1A; Table 1).

**TABLE 1:** Cohort descriptions

VICC-1	
Response	
No NAC	21
pCR	9
RD-nR	16
RD-R	7
Receptor status	
TNBC	17
HR^+^HER2^+^	9
HR^+^HER2^−^	19
HR^−^HER2^+^	8
**GeparNuevo**	
Response	
RD	18
pCR	23
TNBC	
Yes	41
No	0
**Instituto Valenciano**	
Metastasis	
Yes	5
No	9
Hormone receptor +	
Yes	14
No	0
**VICC-SD**	
Response	
RD	75
pCR	35
Receptor status	
TNBC	50
HR^+^HER2^+^	6
HR^+^HER2^−^	44
HR^−^HER2^+^	10

## Materials and Methods

### Patients

For all cohorts, data use was approved by the relevant ethics committee, Institutional Review Board, and national competent authority and adheres to the ethical principles of the Declaration of Helsinki. Written informed consent was obtained for all patients. For the VICC-1 cohort, clinical and pathologic data were retrieved from medical records under an institutionally approved protocol (VICC IRB 030747). For the VICC-SD cohort, clinical and pathologic data were retrieved from the deidentified synthetic derivative medical record under an institutionally approved protocol (VICC IRB 202207). First, all female patients with International Classification of Diseases, 9th/10th Revision, Clinical Modification (ICD-9/10-CM) billing codes for malignant neoplasm of breast were identified. Next, from this cohort, patients with at least one breast cancer surgical procedure using Current Procedural Terminology (CPT) codes and exposure to cyclophosphamide and doxorubicin within 12 months preceding the surgery date were automatically selected. The ICD and CPT codes used for this purpose are listed in the Supplementary Tables S2 and S3, respectively. Finally, the electronic health records of the selected patients were manually reviewed to extract the VICC-SD cohort used in this study. To guide the manual chart review process, additional clinical information was automatically extracted from the SD. This includes breast cancer surgery dates, dates when patients received cyclophosphamide and doxorubicin, laboratory measurements, other chemotherapy or cytokine-related medications and date of administration, pathology reports and operative notes. The GeparNuevo cohort consisted of patients from the placebo arm of the GeparNuevo trial (registration number NCT02685059; ref. 3). The Instituto Valenciano de Oncología cohort consisted of hormone receptor–positive (HR^+^) HER2^−^ patients treated with neoadjuvant chemotherapy. All data use was approved by the relevant ethics committees.

### RNA Sequencing

RNA was extracted from 0.5–2 mL of whole blood or processed peripheral blood mononuclear cells (PBMC) using the Promega Maxwell RSC simplyRNA Blood kit (AS1380, Promega). Total RNA quality was assessed using the 2200 TapeStation (Agilent). Library preparation was done with a ribo-depletion total RNA library preparation kit and 150 bp paired-end sequencing was performed on the Illumina NovaSeq 6000 targeting an average of 10M reads per sample. Quality control was evaluated at different levels, including RNA quality, raw read data, alignment, and gene expression. Raw RNA sequencing (RNA-seq) paired ends were mapped to the human reference genome hg19 using STAR 2.7.3. Raw reads count matrix was calculated by featureCounts and used for downstream analysis. A total of 25 genes were removed from the RNA-seq analysis and transcripts-per-million calculation due to overrepresentation. These genes represent red blood cell contamination of the PBMC. The removed genes are: “RN7SL1,” “RN7SL2,” “HBA1,” “HBA2,” “HBB,” “HBQ1,” “HBZ,” “HBD,” “HBG2,” “HBE1,” “HBG1,” “HBM,” “MIR3648-1,” “MIR3648-2,” “AC104389.6,” “AC010507.1,” “SLC25A37,” “SLC4A1,” “NRGN,” “SNCA,” “BNIP3L,” “EPB42,” “ALAS2,” “BPGM,” “OSBP2.” DESeq2 was used to identify differentially expressed genes and apeglm was used for log fold change reduction (4, 5). Gene-set enrichment analysis (GSEA) was used to identify pathways using the Molecular Signatures Database hallmark gene sets (6, 7). CIBERSORTx was used in relative mode with 500 permutations and the LM22 reference matrix (8). Simplified cell-type categories were collapsed as follows: CD4 T cells = Memory activated CD4 T cells + memory resting CD4 T cells + naïve CD4 T cells + regulatory T cells. B cells = Naïve B cells + Memory B cells + plasma cells. Natural killer (NK) cells = activated NK cells + resting NK cells. Other Myeloid = M0 macrophages + M1 macrophages + M2 macrophages + activated dendritic cells + resting dendritic cells. Notably, these populations were very low abundance as these are all cell types not commonly seen in the peripheral blood. Granulocytes = activated mast cells + resting mast cells + eosinophils + neutrophils.

### NanoString nCounter Analysis

Gene expression was assessed on the GeparNuevo cohort using a custom NanoString Elements panel to measure peripheral immunologic response score (PIRS) genes according to the manufacturer's standard protocol. Briefly, RNA was extracted from processed PBMC pellets using the Promega Maxwell RSC simplyRNA Blood kit and 50 ng of total RNA was used for input into nCounter hybridizations. Data were normalized according to positive and negative spike-in controls, then endogenous housekeeper controls, and transcript counts were log transformed for downstream analyses. PIRS was calculated as described for RNA-seq data, using Z-scores.

### Statistical Analysis

All statistical analyses were performed in R. Single-cell statistical analyses were calculated in R using the Seurat package (9, 10). Shared nearest neighbors were calculated using the Harmony reduction, and clusters were identified at a resolution of 0.3. Uniform Manifold Approximation and Projection (UMAP) was performed for visualization, and missing values were imputed using ALRA (11). Cell types were assigned to individual cells using SingleR (12). BlueprintEncodeData was used as a reference (13, 14). Heatmaps were generated using the R package Complex Heatmap (15). ROC analyses were done in R using the package precrec (16). *P*-value cutoffs displayed on plots correspond to “ns” equals *P* > 0.05, * equals 0.01 < *P* < 0.05, ** equals 0.001 < *P* < 0.01, *** equals 0.0001 < *P* < 0.001, **** equals *P* < 0.0001.

### Data Availability

The datasets generated during and analyzed during the current study have been deposited in NCBI's Gene Expression Omnibus (GEO) and are accessible through GEO Series accession number GSE201085 (https://www.ncbi.nlm.nih.gov/geo/query/acc.cgi?acc=GSE201085). Code used to generate figures can be found at: https://github.com/MLAxelrod/BC_Blood_Monocytes.

## Results

RNA-seq was performed on whole blood of 53 patients with breast cancer after completion of NAC (if received) and prior to definitive surgery (Fig. 1A; *n* = 23 RD) nine pathologic complete response (pCR), 21 no NAC; Table 1). We stratified patients with RD by whether they experienced a breast cancer recurrence (RD-R) or remained free of recurrence for at least 3 years (RD-nR). Follow-up time was at least 3 years for all patients (mean 8.9 years, max 14.5 years), which covers the time period at highest risk for recurrence (17–19). Using DeSeq, we identified 1,238 (FDR corrected *q*-value <0.1) differentially expressed genes between pCR and RD samples (Supplementary Table S1; ref. 4). Using GSEA, we collapsed differentially expressed genes into pathways using the Molecular Signatures Database hallmark gene sets (6, 7). Hallmark IFN Gamma Response [*q*-value <0.0001; normalized enrichment score (NES) = 3.32], Hallmark IFN Alpha Response (*q*-value <0.0001; NES = 3.14), and Hallmark complement (*q*-value = 0.000111; NES = 2.29) pathways were significantly enriched in the blood of patients experiencing pCR compared with those with RD (Fig. 1B). No pathways were statistically significantly upregulated in RD samples relative to pCR samples. To evaluate the genes involved in these pathways, we identified the leading-edge genes from each pathway (IFNγ = 49 genes; IFNα = 26 genes; complement = 15 genes) and selected only the unique genes (*n* = 60 genes). There is strong, uniform upregulation of many of these genes in many of the pCR samples, regardless of triple-negative breast cancer (TNBC) status (Supplementary Fig. S1a). We combined expression of these genes into an IFN/complement score, calculated as sum of z-scores divided by number of genes in the signature (*n* = 60 genes). We compared expression of the IFN/complement score with a previously published 8-gene cytotoxic score (*FGFBP2* + *GNLY* + *GZMB* + *GZMH* + *NKG7* + *LAG3* + *PDCD1* − *HLA-G*; ref. 2). No genes overlapped between the two signatures. Samples with the highest expression of the IFN/complement had low expression of the cytotoxic score and tended to be pCR samples. Conversely, those with highest expression of the cytotoxic score tended to have low expression of the IFN/complement score and be RD samples (Fig. 1C). A combination PIRS of IFN/complement score minus cytotoxic score had improved predictive power compared with either signature alone (*P* = 0.006 for pCR vs. RD-R, *P* = 0.01 for pCR vs. RD-nR, Wilcoxon tests with FDR corrections; Fig. 1D). The same trends were observed when examining TNBC, ER^+^, or HER2^+^ only patients (Supplementary Fig. S1b). To examine which cell types predominately express each signature, we used single-cell RNA-seq data from whole PBMCs from 2 patients with breast cancer post-NAC, prior to surgery (2). These 2 patients were not included in any other analyses. Expression of the cytotoxic score was the highest in CD8^+^ T cells and NK cells, while the IFN/complement score was the highest in a subset of monocytes. There was very little coexpression of the signatures across cells (Fig. 1E; Supplementary Fig. S1c).

**FIGURE 2:**
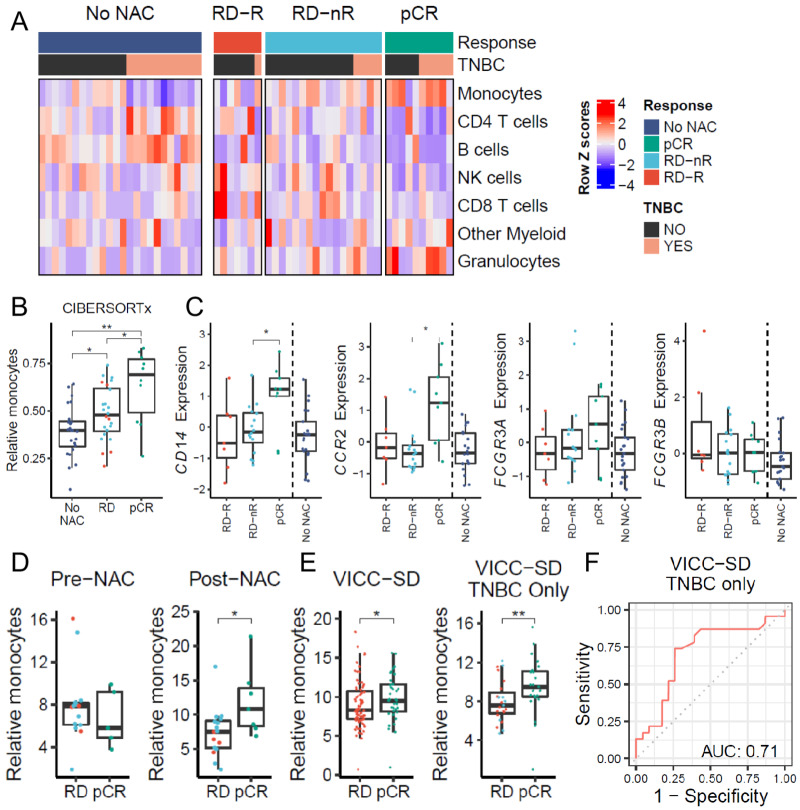
Monocytes are most abundant in blood of patients with good outcomes following NAC. **A,** Heatmap showing row standardized (z-score) of CIBERSORTx inferred cell type abundance. Most abundant cell types are listed first. **B,** CIBERSORTx monocyte values, stratified by outcome. **C,** Row standardized (z-score) expression of key monocyte related genes in VICC-1 RNA-seq cohort. **D,** Clinical lab values for relative monocytes pre- and post-NAC. **E,** Relative monocytes in full and TNBC only synthetic derivative cohort. **F,** ROC curve for differentiating pCR versus RD in the VICC-SD TNBC only cohort. Box plots show the median, first and third quartiles. *P* values represent one-tailed Wilcox tests (FDR corrected where applicable).

Given the partitioning of the gene expression scores into cell types, we next aimed to identify whether there were differences in cell type abundances between the outcome groups. CIBERSORTx was used to deconvolute relative cell-type abundance from the RNA-seq data (ref. 8; Fig. 2A). Relative mode normalizes all cell fractions of the cell types in the signature matrix to 100%. Relative monocyte abundance was highest in samples with pCR, intermediate in those with RD, and lowest in samples not receiving NAC (*P* = 0.003 for No NAC vs. pCR, *P* = 0.028 for No NAC vs. RD, *P* = 0.028 for pCR vs. RD, Wilcox tests with FDR corrections for multiple comparisons; Fig. 2B). Naïve B cells were also statistically significantly different across groups, being highest in no NAC samples and lowest in pCR samples (*P* = 0.0009 for no NAC vs. pCR, *P* = 0.0008 for no NAC vs. RD, *P* = 0.043 for pCR vs. RD, Wilcox tests with FDR corrections for multiple comparisons; Supplementary Fig. S2a). However, only monocytes followed the trend of increases from no NAC to RD-R to RD-nR to pCR (Supplementary Fig. S2b). The trend of higher monocytes in patients with pCR relative to RD was also observed when examining TNBC, ER^+^, or HER2^+^ only patients (Supplementary Fig. S2c).

Monocytes comprise a heterogenous population and differing monocyte subpopulations have been associated with both good and poor outcomes in cancer (20–26). The majority of circulating monocytes have a classical phenotype, characterized by CD14 expression and lack of CD16 expression (20). We found that gene expression of classical monocyte-associated genes (i.e., *CD14 P* = 0.05 *CCR2 P* = 0.013, for pCR vs. RD-nR two-tailed Wilcox tests with FDR corrections for multiple comparisons) was significantly higher in the blood of patients with pCR, whereas there was no significant difference in expression of genes associated with non-classical monocytes (i.e., *FCGR3A*, *FCGR3B* which are the genes encoding CD16; Fig 2C).

The routine nature of clinically measuring total monocytes made monocyte values an intriguing metric for further study. We reviewed electronic medical records and extracted monocyte values from complete blood counts for patients receiving NAC in this cohort. A total of 23 of 32 (72%) of NAC-treated patients (*n* = 7 pCR, 12 RD-nR, 4 RD-R) had a complete blood count with differential (which includes monocyte values) in the 30-day interval prior to surgery (following completion of NAC), indicating the commonality of collecting this information clinically. Clinically measured relative monocyte values are reported as percent of circulating leukocytes. Clinically measured monocyte values were significantly positively correlated with monocyte values inferred by CIBERSORTx (*R* = 0.51, *P* = 0.012; Supplementary Fig. S2d), even though monocyte values were not always collected on the same day as the blood for RNA-seq (though in the same 30-day window). Post-NAC, but not pre-NAC, clinically measured monocytes were significantly higher in patients with pCR compared with those with RD (*P* = 0.0197, one-tailed Wilcoxon for RD vs. pCR; Fig. 2D; *P* = 0.018 one-tailed Wilcoxon with FDR correction for RD-R vs. pCR; Supplementary Fig. S2E). The change in monocytes from pre- to post-NAC was not statistically significantly different for patients with RD or pCR, though the monocytes increased from pre- to post-NAC for most patients with pCR (Supplementary Fig. S2f).

Next, we sought to assess whether monocyte abundance was associated with outcome in independent cohorts. Higher monocytes were also seen with pCR in an additional cohort of 41 patients with TNBC (*n* = 18 RD, 23 pCR; placebo arm of the GeparNuevo study; Table 1), though this association was not statistically significant (*P* = 0.0638 for absolute monocyte counts, *P* = 0.186 for relative monocyte frequencies, one-tailed Wilcoxon; Supplementary Fig. S3a; ref. 3). In this TNBC only dataset, PIRS, measured by NanoString, was not associated with outcome, indicating independence of monocytes and PIRS measurements (Supplementary Fig. S3b). In an additional independent cohort of 14 patients with HR^+^ HER2^−^ breast cancer from the Instituto Valenciano de Oncología, monocytes tended to be higher in patients without metastatic recurrence, with at least 4 years of follow-up time for each patient (*P* = 0.0949, one-tailed Wilcoxon; *n* = 5 with metastasis, 9 without metastasis; Supplementary Fig. S3c; Table 1). Using a deidentified medical record database called the synthetic derivative (SD), we identified 110 breast cancer patients (VICC-SD; *n* = 35 pCR, 75 RD; Table 1) who had been treated with NAC, had a breast surgery, and had a monocyte value within 30 days prior to surgery. In the VICC-SD cohort, relative frequencies of monocytes were statistically significantly higher in patients with pCR compared with those with RD (*P* = 0.037 one-tailed Wilcoxon; Fig. 2E). This effect was more pronounced when considering only the patients with TNBC (*P* = 0.0074, one-tailed Wilcoxon; *n* = 50), which may be reflective of underlying TNBC-specific biology, or the more uniform treatment options for TNBC (chemotherapy rather than targeted therapy agents; *P* = 0.046 for RD-R vs. pCR, one-tailed Wilcoxon with FDR correction; Supplementary Fig. S3d). In all cohorts, patients who had received cytokine-support products (i.e., GCSF) in the 30-day window prior to surgery were excluded from analysis as this may affect monocyte counts. To summarize our findings, we performed ROC analyses on all key findings. AUC for ROC analyses are summarized in Table 2. Where possible, we calculated the AUC for differentiating pCR versus RD as well as overall good outcome (pCR and RD-nR) with poor outcome (RD-R), which may be more clinically useful. A representative ROC curve for using monocytes to differentiate pCR versus RD in the VICC-SD TNBC only cohort is shown in Fig. 2F. Taken together, these data suggest that higher blood monocyte levels post-NAC may be indicative of superior outcomes in patients with breast cancer.

**TABLE 2:** AUC analyses

Metric	Method	Cohort	AUC (pCR vs. RD)	AUC (no recurrence vs. recurrence)
PIRS	RNA sequencing	VICC-1	0.86	0.75
PIRS	NanoString	GeparNuevo	0.41	NA
Monocytes	CIBERSORTx (RNA sequencing)	VICC-1	0.74	0.70
Monocytes	Clinical	VICC-1	0.65	0.68
Monocytes	Clinical	VICC-SD	0.61	NA
Monocytes	Clinical	VICC-SD TNBC only	0.71	0.66
Monocytes	Clinical	Instituto Valenciano	NA	0.73 * (metastasis vs. no)
Monocytes	Clinical	GeparNuevo	0.64 (absolute); 0.58 (relative)	NA

## Conclusions/Discussion

Peripheral blood gene expression scores and cell type abundance may be useful biomarkers of NAC response and outcomes in breast cancer. We identified an immunologic gene signature (PIRS) that was highest in patients with the best outcomes (pCR) and lowest in those with the worst outcome (RD with recurrence). However, PIRS was not associated with outcome in a separate cohort of TNBC only patients. There are several possible reasons for this difference including that the GeparNuevo cohort consisted of the control arm of a clinical trial and these patients received a more uniform chemotherapy regimen (nanoparticle albumin-bound paclitaxel followed by epirubicin and cyclophosphamide). Ten patients from this cohort were excluded from our analyses due to proximal treatment with cytokine-support products (i.e., GCSF), relative to only one excluded in the VICC-1 cohort. The VICC-1 patients were treated as routine clinical standard of care and included patients of all histologic subtypes. Additional studies are needed to test whether PIRS or other gene expression scores may be useful biomarkers in patients with breast cancer. Additional studies will also be needed to identify and standardize appropriate cutoffs for gene expression scores.

Higher peripheral monocytes, a standard clinical assay performed on most patients with breast cancer, was associated with improved patient outcomes (pCR or lack of recurrence). The association of higher monocytes with improved outcomes was observed in four independent breast cancer patient cohorts, though this difference was only statistically significant (*P* < 0.05) in two of four cohorts. Circulating monocyte subpopulations have previously been associated with both good and poor outcomes in several cancer types (21, 24, 27, 28). Interestingly, a prior study in patients with breast cancer showed that a stronger IFNγ response in monocytes was associated with lack of relapse, in line with our results (22). Several studies have also seen an increase in circulating monocytes following chemotherapy in breast and other cancer types, and this increase has variably been statistically or numerically associated with better outcomes (23, 26, 28). Given that blood monocytes may increase following chemotherapy in multiple tumor types and in our data higher monocytes are associated with better outcomes regardless of breast cancer histologic subtype, we hypothesize that the increase in monocytes may represent postchemotherapy hematopoietic regeneration, which is likely to be more reflective of the patient's immune and hematopoietic system than tumor-intrinsic biology. However, this idea remains to be tested. Additional efforts are needed to explore whether there might be a causal link between chemotherapy induced monocyte mobilization and improved response. As blood is sampled following completion of NAC, it is unknown how stage of disease, nodal status, or other clinicopathologic variables may affect gene expression or cell-type abundances.

Our study has several important limitations. Our study is retrospective and no pretreatment blood samples were available to study changes in gene expression over the course of treatment. We were also limited by differences in data availability for each cohort and differences in patient characteristics by cohort. An important confounding factor is the use of GMCSF products. All patients receiving GMCSF products were excluded from our analyses, due to high likelihood of this intervention changing cell-type abundance and gene expression. However, this exclusion criteria may have introduced bias into our results by excluding patients who required cytokine supportive therapy. We were also limited by size of the available cohorts matching our inclusion criteria, leading some of our analyses to be underpowered.

Overall, we hypothesize that the association of higher monocytes with good outcomes reflects robust hematopoietic regeneration following chemotherapy and indicates good overall health which may be independently related to good outcomes. However, further studies are needed to test this hypothesis. Studies using murine model systems will be able to more directly test whether peripheral monocytes play a mechanistic role in good outcomes or are an independent biomarker, without a mechanistic role.

Taken together, these results suggest that peripheral blood biomarkers following NAC may be useful in predicting long-term outcome. Future work will explore the utility of peripheral blood biomarkers in predicting immunotherapy response.

## Supplementary Material

Supplementary Table 1 Differentially expressed genes between pCR and RD samples.

Supplementary Tables 2 and 3 ICD and CPT codes used for synthetic derivative analyses.

Supplementary Figure Legends Legends for Supplementary Figures 1-3

Supplementary Figures 1-3 Supplementary Figure 1. Expression of immune related genes in the peripheral blood is associated with good outcome following NAC. Supplementary Figure 2. Monocytes are most abundant in blood of patients with good outcomes following NAC. Supplementary Figure 3. Monocytes are most abundant in blood of patients with good outcomes following NAC.
