# Supplementary material for: Peripheral Blood Monocyte Abundance Predicts Outcomes in Patients with Breast Cancer
Source: Cancer Res Commun. 2022 May 4;2(5):286–92. doi: 10.1158/2767-9764.CRC-22-0023 (PMC9604512; doi:10.1158/2767-9764.CRC-22-0023)
Supplement: Supplementary Tables 2 and 3 — ICD and CPT codes used for synthetic derivative analyses. [file crc-22-0023-s02.docx]

**Supplementary Table 2** ICD codes

| **Code** | **Description** |
| --- | --- |
| ICD9CM |  |
| 174 | Malignant neoplasm of female breast |
| 174.0 | Malignant neoplasm of nipple and areola of female breast |
| 174.1 | Malignant neoplasm of central portion of female breast |
| 174.2 | Malignant neoplasm of upper-inner quadrant of female breast |
| 174.3 | Malignant neoplasm of lower-inner quadrant of female breast |
| 174.4 | Malignant neoplasm of upper-outer quadrant of female breast |
| 174.5 | Malignant neoplasm of lower-outer quadrant of female breast |
| 174.6 | Malignant neoplasm of axillary tail of female breast |
| 174.8 | Malignant neoplasm of other specified sites of female breast |
| 174.9 | Malignant neoplasm of breast (female), unspecified |
| ICD10CM |  |
| C50 | Malignant neoplasm of breast |
| C50.0 | Malignant neoplasm of nipple and areola |
| C50.01 | Malignant neoplasm of nipple and areola, female |
| C50.011 | Malignant neoplasm of nipple and areola, right female breast |
| C50.012 | Malignant neoplasm of nipple and areola, left female breast |
| C50.019 | Malignant neoplasm of nipple and areola, unspecified female breast |
| C50.1 | Malignant neoplasm of central portion of breast |
| C50.11 | Malignant neoplasm of central portion of breast, female |
| C50.111 | Malignant neoplasm of central portion of right female breast |
| C50.112 | Malignant neoplasm of central portion of left female breast |
| C50.119 | Malignant neoplasm of central portion of unspecified female breast |
| C50.2 | Malignant neoplasm of upper-inner quadrant of breast |
| C50.21 | Malignant neoplasm of upper-inner quadrant of breast, female |
| C50.211 | Malignant neoplasm of upper-inner quadrant of right female breast |
| C50.212 | Malignant neoplasm of upper-inner quadrant of left female breast |
| C50.219 | Malignant neoplasm of upper-inner quadrant of unspecified female breast |
| C50.3 | Malignant neoplasm of lower-inner quadrant of breast |
| C50.31 | Malignant neoplasm of lower-inner quadrant of breast, female |
| C50.311 | Malignant neoplasm of lower-inner quadrant of right female breast |
| C50.312 | Malignant neoplasm of lower-inner quadrant of left female breast |
| C50.319 | Malignant neoplasm of lower-inner quadrant of unspecified female breast |
| C50.4 | Malignant neoplasm of upper-outer quadrant of breast |
| C50.41 | Malignant neoplasm of upper-outer quadrant of breast, female |
| C50.411 | Malignant neoplasm of upper-outer quadrant of right female breast |
| C50.412 | Malignant neoplasm of upper-outer quadrant of left female breast |
| C50.419 | Malignant neoplasm of upper-outer quadrant of unspecified female breast |
| C50.5 | Malignant neoplasm of lower-outer quadrant of breast |
| C50.51 | Malignant neoplasm of lower-outer quadrant of breast, female |
| C50.511 | Malignant neoplasm of lower-outer quadrant of right female breast |
| C50.512 | Malignant neoplasm of lower-outer quadrant of left female breast |
| C50.519 | Malignant neoplasm of lower-outer quadrant of unspecified female breast |
| C50.6 | Malignant neoplasm of axillary tail of breast |
| C50.61 | Malignant neoplasm of axillary tail of breast, female |
| C50.611 | Malignant neoplasm of axillary tail of right female breast |
| C50.612 | Malignant neoplasm of axillary tail of left female breast |
| C50.619 | Malignant neoplasm of axillary tail of unspecified female breast |
| C50.8 | Malignant neoplasm of overlapping sites of breast |
| C50.81 | Malignant neoplasm of overlapping sites of breast, female |
| C50.811 | Malignant neoplasm of overlapping sites of right female breast |
| C50.812 | Malignant neoplasm of overlapping sites of left female breast |
| C50.819 | Malignant neoplasm of overlapping sites of unspecified female breast |
| C50.9 | Malignant neoplasm of breast of unspecified site |
| C50.91 | Malignant neoplasm of breast of unspecified site, female |
| C50.911 | Malignant neoplasm of unspecified site of right female breast |
| C50.912 | Malignant neoplasm of unspecified site of left female breast |
| C50.919 | Malignant neoplasm of unspecified site of unspecified female breast |

**Supplementary Table 3** CPT codes

| **Code** | **Description** |
| --- | --- |
| 19125 | Excision of breast lesion identified by preoperative placement of radiological marker, open; single lesion |
| 19126 | Excision of breast lesion identified by preoperative placement of radiological marker, open; each additional lesion separately identified by a preoperative radiological marker (List separately in addition to code for primary procedure) |
| 19140 | Mastectomy for gynecomastia |
| 19160 | Mastectomy, partial |
| 19162 | Mastectomy, partial; with axillary lymphadenectomy |
| 19180 | Mastectomy, simple, complete |
| 19182 | Mastectomy, subcutaneous |
| 19200 | Mastectomy, radical, including pectoral muscles, axillary lymph nodes |
| 19220 | Mastectomy, radical, including pectoral muscles, axillary and internal mammary lymph nodes (Urban type operation) |
| 19240 | Mastectomy, modified radical, including axillary lymph nodes, with or without pectoralis minor muscle, but excluding pectoralis major muscle |
| 19301 | Mastectomy, partial (eg, lumpectomy, tylectomy, quadrantectomy, segmentectomy) |
| 19306 | Mastectomy, radical, including pectoral muscles, axillary and internal mammary lymph nodes (Urban type operation) |
| 19307 | Mastectomy, modified radical, including axillary lymph nodes, with or without pectoralis minor muscle, but excluding pectoralis major muscle |
