# Supplementary material for: Peripheral Blood Monocyte Abundance Predicts Outcomes in Patients with Breast Cancer
Source: Cancer Res Commun. 2022 May 4;2(5):286–92. doi: 10.1158/2767-9764.CRC-22-0023 (PMC9604512; doi:10.1158/2767-9764.CRC-22-0023)
Supplement: Supplementary Figure Legends — Legends for Supplementary Figures 1-3 [file crc-22-0023-s03.docx]

Supplementary Figure 1: *Expression of immune related genes in the peripheral blood is associated with good outcome following NAC*. A) Heatmap showing row standardized (z-score) gene expression for leading edge IFN/Complement genes (n=60 genes). B) PIRS for TNBC only, ER+ only or HER2+ only patients in the VICC-1 cohort, stratified by outcome. C) Violin plots showing the distribution of PIRS for each cell type in single cell sequencing data. Box plots show the median, first and third quartiles.

Supplementary Figure 2: *Monocytes are most abundant in blood of patients with good outcomes following NAC.* A) Naïve B cells, as measured by CIBERSORTx, stratified by response to NAC. B) Monocytes and naïve B cells, as measured by CIBERSORTx, stratified by response and recurrence. C) CIBERSORTx monocyte values, stratified by outcome and breast cancer subtype. D) Correlation between clinically measured relative monocytes extracted from the electronic health record and monocytes inferred by CIBERSORTx. E) Post-NAC clinically measured monocytes, stratified by response and recurrence. F) Change in clinically measured relative monocytes from pre- to post-NAC stratified by patients with RD or pCR. Box plots show the median, first and third quartiles. P-values represent one-tailed Wilcox tests (FDR corrected where applicable).

Supplementary Figure 3: *Monocytes are most abundant in blood of patients with good outcomes following NAC.* A) Relative and absolute monocytes in TNBC cohort from GeparNuevo placebo arm, stratified by NAC outcome. B) Peripheral immunologic response score (PIRS) in GeparNuevo TNBC cohort, as measured by NanoString. C) Relative monocytes in the Instituto Valenciano HR+ cohort, stratified by metastasis. D) Relative monocytes in VICC-SD cohort groups, by breast cancer subtype. Box plots show the median, first and third quartiles. P-values represent one-tailed Wilcox tests (FDR corrected where applicable).
