# Supplementary material for: Peripheral Blood Monocyte Abundance Predicts Outcomes in Patients with Breast Cancer
Source: Cancer Res Commun. 2022 May 4;2(5):286–92. doi: 10.1158/2767-9764.CRC-22-0023 (PMC9604512; doi:10.1158/2767-9764.CRC-22-0023)
Supplement: Supplementary Figures 1-3 — Supplementary Figure 1. Expression of immune related genes in the peripheral blood is associated with good outcome following NAC. Supplementary Figure 2. Monocytes are most abundant in blood of patients with good outcomes following NAC. Supplementary Figure 3. Monocytes are most abundant in blood of patients with good outcomes following NAC. [file crc-22-0023-s04.pptx]

## Slide 1
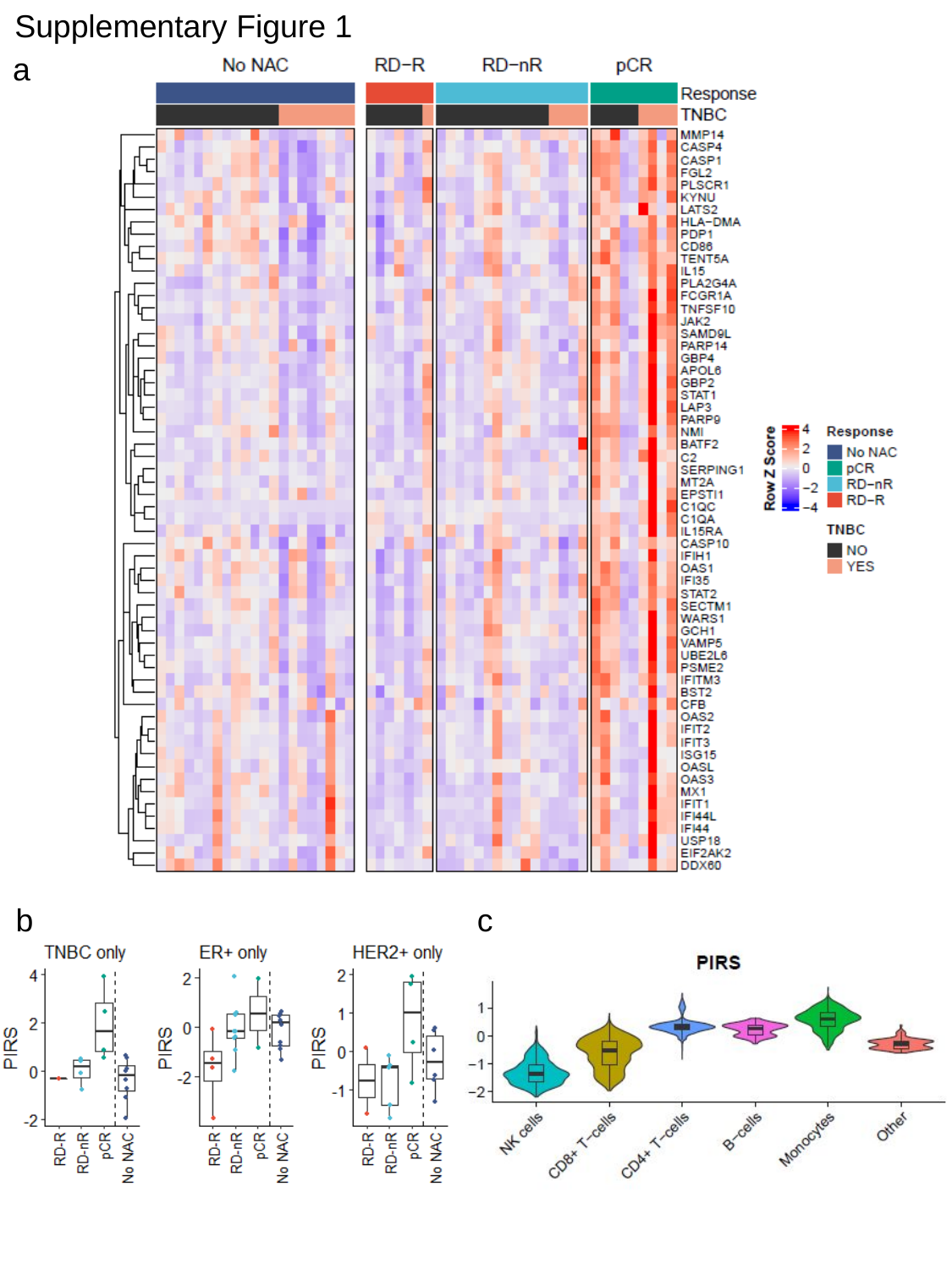

Supplementary Figure 1
a
b
c

## Slide 2
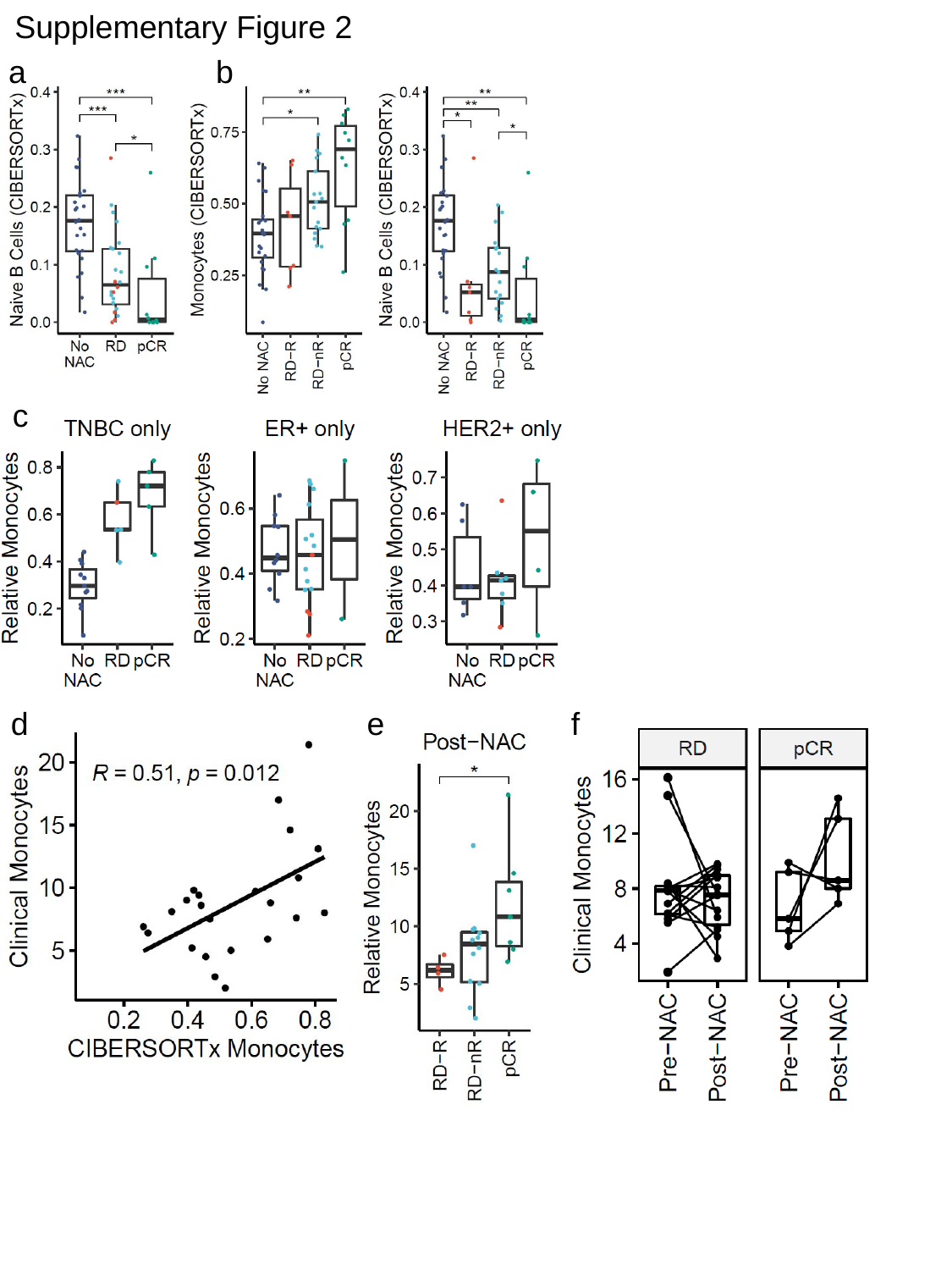

Supplementary Figure 2
a
b
c
d
e
f

## Slide 3
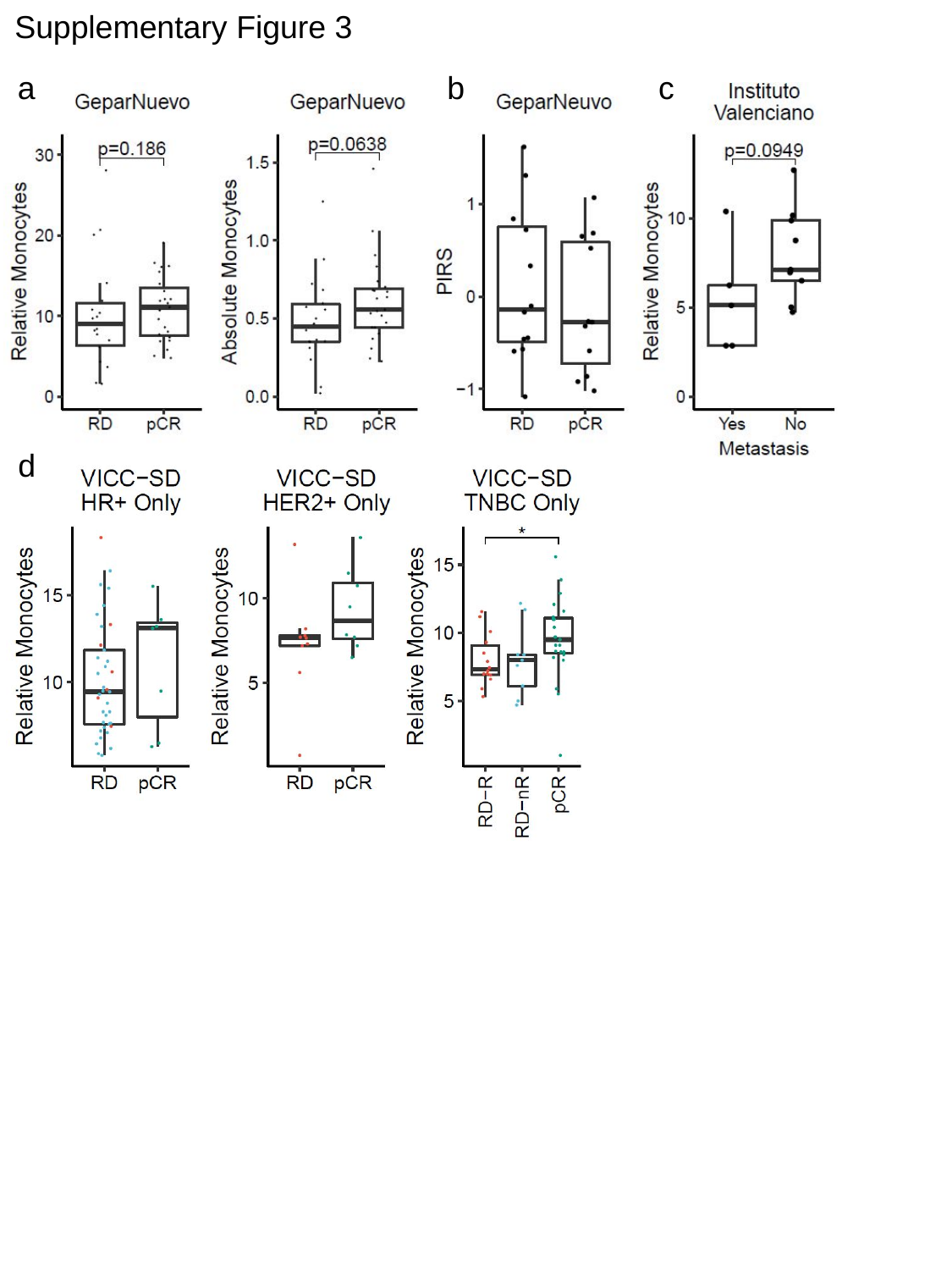

Supplementary Figure 3
a
b
c
d
